# Case Report: Dramatic response to entritinib in a patient with gastrointestinal stromal tumor positive for *NTRK3* fusion

**DOI:** 10.3389/fonc.2025.1588950

**Published:** 2025-08-18

**Authors:** Guomin Dong, Pengyu Han, Zhiyun Zhang, Qian Ge, Jian Jiang, Suoni Li, Jiequn Ma, Jie Bai, Hui Wei, Zheng Zhao

**Affiliations:** ^1^ The First Clinical Medical College, Shaanxi University of Chinese Medicine, Xianyang, China; ^2^ Graduate School, Xi’an Medical University, Xi’an, Shaanxi, China; ^3^ Department of Internal Medicine, Shaanxi Provincial Cancer Hospital, Xi’an, Shaanxi, China

**Keywords:** gastrointestinal stromal tumor, acquired resistance, next-generation sequencing, NTRK3 fusion, ctDNA dynamics predict

## Abstract

Gastrointestinal stromal tumors (GISTs) are the most common mesenchymal tumors of the gastrointestinal tract, with proto-oncogene, receptor tyrosine kinase (c-kit), or PDGFRα mutations detected in around 85% of cases. GISTs without c-kit or platelet-derived growth factor receptor alpha (PDGFRα) mutations are considered wild-type (WT). Recently, some molecular alterations, including neurotrophic tyrosine receptor kinase (NTRK) fusions, have been reported in very few cases of WT GISTs. This novel finding opens the window for the use of tropomyosin receptor kinase (TRK) inhibitor therapy in these subtypes of GIST. In this case report, we present a rare *NTRK3* fusion gastrointestinal stromal tumor (GIST) in a female patient with significant response to entrectinib. The patient was initially diagnosed with a giant gastric GIST (approximately 20.6cm×12.1cm×28.0m in size) showing classic immunohistochemical features (CD117+/DOG1+) on immunohistochemistry. After neoadjuvant imatinib therapy (400 mg/day), partial response was achieved with tumor shrinkage to 14.1cm×7.6cm×15.5cm, followed by radical surgery. Postoperative pathology confirmed high-risk GIST (ypT4N0), with genetic testing revealing a KIT exon11 deletion mutation (p.K558_V560del, VAF 63.80%). Continued oral imatinib adjuvant therapy was initiated. In 2024, disease progression was observed with residual KIT mutation (VAF 1.10%) and new-onset *ETV6:NTRK3* fusion (VAF 35.29%) detected by circulating tumor DNA (ctDNA) analysis. Switching to entrectinib (600 mg/day) achieved partial imaging response within 4 weeks (tumor reduction of approximately 27%), with complete clearance of dual mutations observed in ctDNA after 3 months. The patient maintained sustained response without adverse events during final follow-up. This case highlights the breakthrough efficacy of TRK inhibitors in treating NTRK-fusion GIST and confirms the critical value of liquid biopsy in monitoring drug resistance mechanisms and guiding precision treatment.

## Introduction

Gastrointestinal stromal tumor (GIST), the most common mesenchymal malignancy of the gastrointestinal tract, has been classified into five major molecularly subtypes through next-generation sequencing (NGS) including classical *KIT/PDGFRA*-mutant, *SDH*-deficient, *NF1*-assoicated, *BRAF*-mutant, and *NTRK3*-fusion GISTs (1). Notably, the *NTRK3* fusion have emerged as rare but clinically significant molecular alternations defining a distinct subgroup of GISTs (2). Therefore, elucidating the clinicopathological characteristics, diagnostic approaches, and therapeutic implications regarding *NTRK3* rearrangements hold critical value for advancing precision oncology. Herein, we present a case of a 60-year-old female diagnosed with an *NTRK3* fusion, following we also include a comprehensive review of the current literature regarding this rare molecular entity.

## Case description

**Figure 1:**
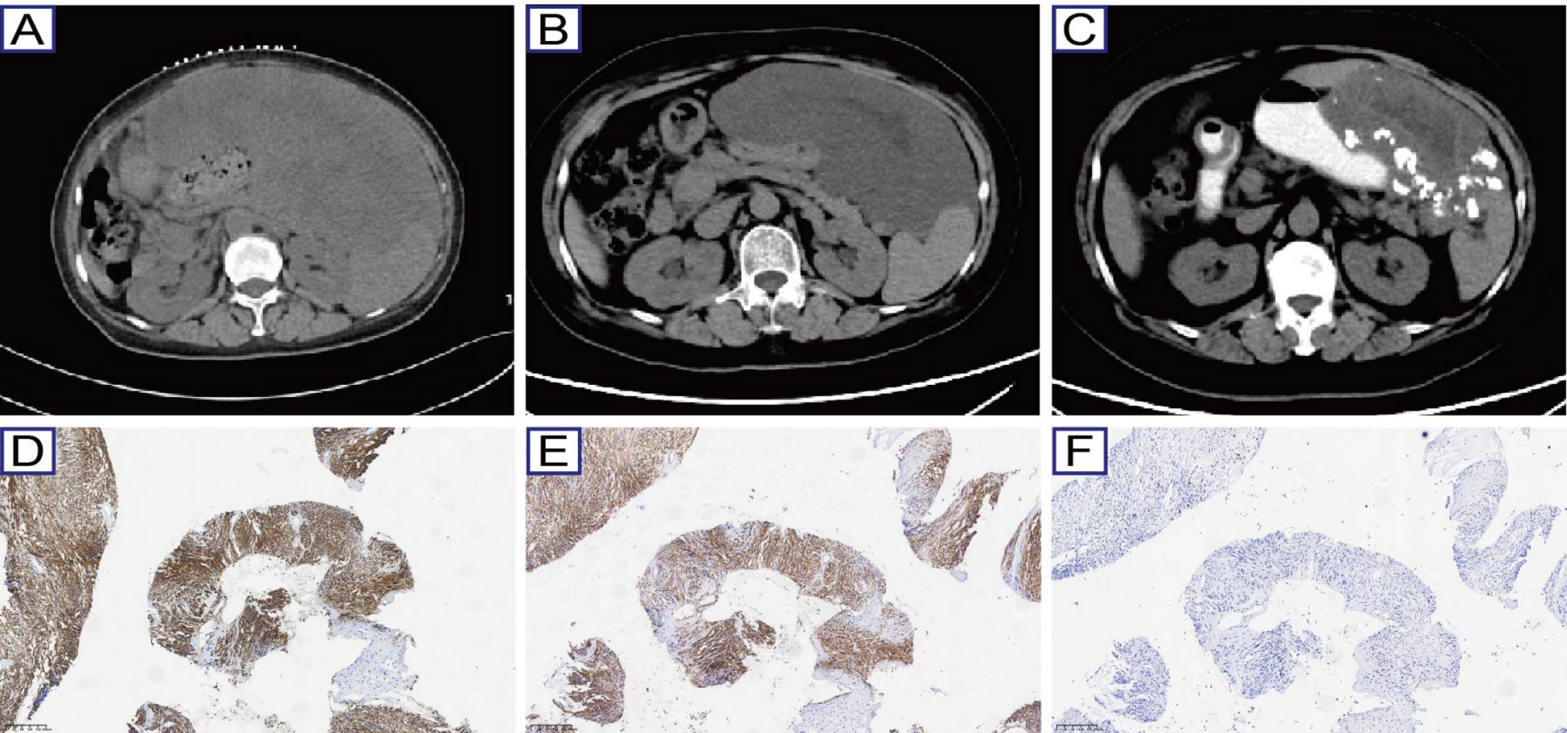
Imaging and pathological characteristics of *NTRK3*-fusion GIST case. **(A)** Baseline contrast-enhanced CT (August 2018) demonstrates a massive left abdominal mass (20.6 cm×12.1 cm×28.0 cm) with infiltrative borders (arrows) involving the gastric fundus and pancreatic tail; **(B)** Post-neoadjuvant therapy imaging (October 2018) reveals partial response with tumor reduction (19.0 cm×8.8 cm×23.5 cm) and improved margination (arrows); **(C)** Long-term follow-up CT (September 2022) confirms sustained partial response (14.1 cm×7.6 cm×15.5 cm) with persistent gastrotumor communication (dashed circle); **(D-F)** Representative immunohistochemical staining for CD117, CD34 and Ki-67.

A 60-year-old female presented with progressive abdominal distension lasting six months, culminating in admission to our institution on August, 2018. Initial evaluation at a local hospital on August, 2018, revealed a massive left abdominal mass (20.6 cm×12.1 cm×28.0 cm) on contrast-enhanced CT (**Figure 1A**), which showed poorly defined borders with adjacent organs (liver, stomach, pancreas, and bowel) and gastric architectural distortion. Radiological features suggested a malignant stromal tumor with extensive ulceration. Elevated CA-125 (161.60 U/mL) and gastroscopic findings further supported this suspicion, revealing a deep ulcerated lesion at the gastroesophageal junction with pseudodiverticular formation and mucosal irregularity. Biopsy pathology (No. 20185032) confirmed moderate chronic inflammation with acute exacerbation but lacked diagnostic clarity. On August 20, 2018, a subsequent CT-guided core needle biopsy was performed. Immunohistochemical analysis confirmed a GIST phenotypes: CD117(+), DOG1(+), CD34(+), SMA(+), Vimentin(+), Ki-67 (40%), CKpan (-), desmin (-), and S-100 (-) (**Figures 1D–F**). Because the gene sequencing technology for gastrointestinal stromal tumors was still in development at the time of the patient’s onset, the patient refused to undergo genetic testing after full communication. Following multidisciplinary tumor board consensus, neoadjuvant therapy with oral imatinib (400 mg daily) was initiated due to extensive tumor is large and involvement of the gastric wall, pancreas, and spleen. Two months of treatment resulted in volume reduction (19 cm×8.8 cm×23.5 cm) and improved margination (**Figure 1B**). Continued therapy-maintained disease control, with final follow-up on September 24, 2022, demonstrating sustained partial response (14.1 cm×7.6 cm×15.5 cm) and persistent gastrotumor communication (**Figure 1C**).

**Figure 2:**
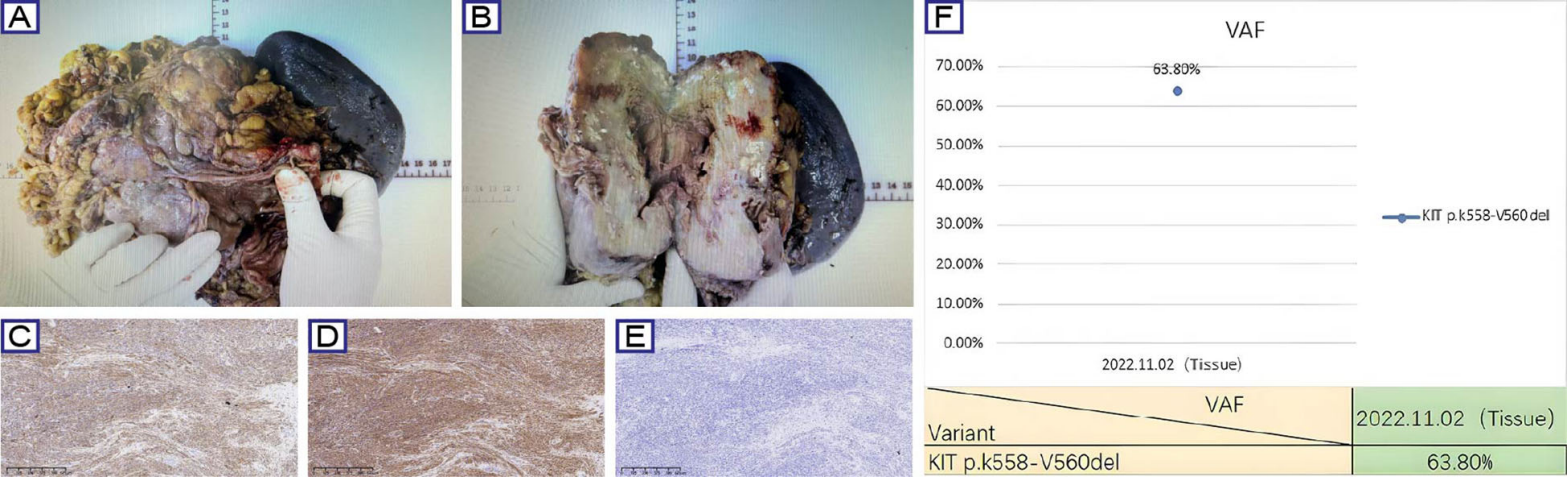
Surgical, histopathological, and molecular characteristics of GIST case. **(A, B)** Gross examination of the resected specimen demonstrates a lobulated mass (19 cm maximal diameter) with hemorrhagic necrosis (asterisk) and fibrous pseudocapsule (arrows). **(C-E)** Representative immunohistochemical staining for CD117, CD34 and Ki-67. **(F)** NGS identified a pathogenic *KIT* exon 11 deletion (p.K558_V560del) with 63.80% variant allele frequency.

In November 2022, the patient received a radical surgery at a tertiary hospital in Shaanxi province, involving proximal gastrectomy, distal pancreatectomy with splenectomy, and adhesiolysis. Histopathological evaluation of the resected specimen (proximal stomach, spleen, and pancreatic tail) demonstrated treatment-induced morphological changes, characterized by extensive hyaline degeneration and multifocal calcifications. The resected tumor measured 19 cm in maximum diameter with a mitotic count of 4/50 HPF, showing infiltrative growth into the splenic parenchyma and pancreatic capsule (R1 resection) (**Figures 2A, B**). Lymph node staging revealed no metastasis (0/25 nodes). The tumor was further classified as ypT4N0 (AJCC 8th), 3b (WHO) and high-risk NIH group. Postoperative immunohistochemical profiling confirmed classic GIST immunophenotype with CD117(+), DOG1(+), CD34(+), SDH-a(+), SDH-β(+), Desmin(-), H-cal desmon(-), S-100(-), SHA(-), and Ki-67 (4%) (**Figures 2C–E**). Importantly, NGS on the tumor samples identified a pathogenic *KIT* exon 11 deletion (p.K558_V560del) with 63.80% variant allele frequency (VAF) (**Figure 2F**). Based on multidisciplinary consensus, adjuvant imatinib therapy (400 mg daily) was recommended. However, the patient did not adhere to regular follow-up after 2022.

**Figure 3:**
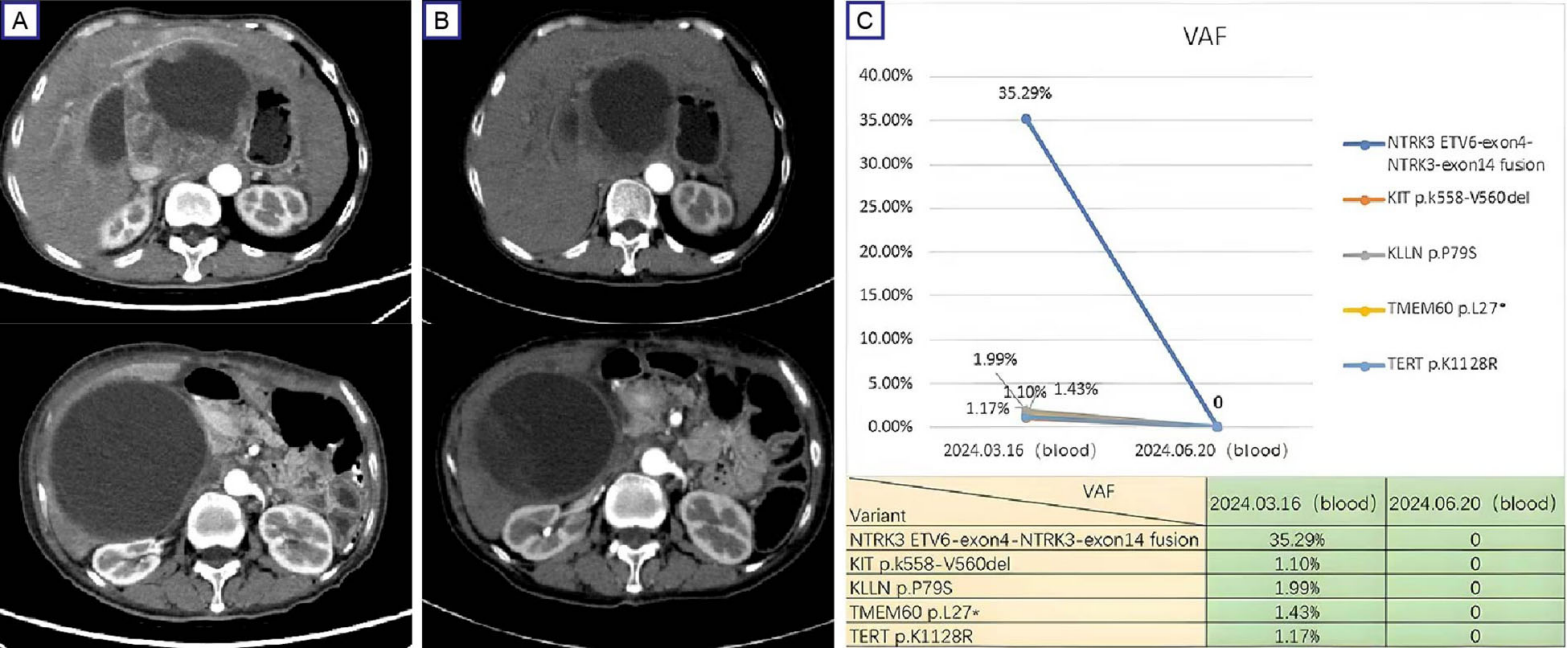
Therapeutic response and mutation dynamics in progressive GIST. **(A)** Pre-treatment contrast-enhanced CT (March 28, 2024) demonstrates a dominant cystic-solid mass (12.6 cm×10.8 cm, arrows) in the hepatogastric space with IVC compression; **(B)** Post-treatment (entrectinib) contrast-enhanced CT (April 25, 2024) shows partial response (RECIST 1.1) with 27% tumor reduction (9.6 cm × 8.4 cm) and improved IVC compression. **(C)** ctDNA profiling reveals clearance of *KIT* exon 11 deletion (p.K558_V560del) and *ETV6:NTRK3* fusion (ETV6 exon4:NTRK3 exon14) following entrectinib therapy. IVC, inferior vena cava.

In March 2024, the patient returned to our hospital with recurrent abdominal distension. Contrast-enhanced CT revealed progressive GIST features include anastomotic soft-tissue thickening, cystic-solid masses and compression changes in the inferior vena cava (**Figure 3A**). As the disease progressed, the patient refused to undergo a repeat biopsy, and Plasma ctDNA analysis was performed after full communication with the family,Plasma ctDNA analysis identified the residual *KIT* mutation (VAF 1.10%) and an recurrent *ETV6:NTRK3* fusion (ETV6 exon4:NTRK3 exon14; VAF 35.29%). Entrectinib therapy (600 mg daily) was initiated on March 28, 2024. Follow-up CT at 4 weeks demonstrated partial response (RECIST 1.1) (**Figure 3B**). Repeat ctDNA profiling in June 2024 confirmed complete clearance of both mutations (**Figure 3C**). The patient remained asymptomatic with sustained radiographic response at final telephone follow-up (October 31, 2024). So far, the patient was continuing entrectinib without reported adverse events.

## Discussion

Gastrointestinal stromal tumors (GISTs) is the most common mesenchymal neoplasms that originated from the interstitial cells of Cajal (ICCs) or their precursors within the gastrointestinal mesenchymal tissue (3). Epidemiological studies indicate a global incidence of 10 to 15 cases per million population, with gastric (60%) and small intestinal (30%) predominance. Less frequent sites include the duodenum (4 to 5%), rectum (2 to 4%), colon (1 to 2%), and esophagus (<1%) (4). Approximately 20 to 30% of GISTs demonstrate malignant behavior, with 5-year survival rates ranging from 35 to 65% for metastatic disease (4). More recent studies also suggest GISTs may also arise in extraintestinal sites such as the mesentery and omentum (5–7). Based on anatomical growth patterns, GISTs are classified into four subtypes: submucosal, intramural, subserosal, and extragastrointestinal (5–7). Most GISTs present as solitary lesions with nonspecific clinical manifestations, often incidentally detected during endoscopic or cross-sectional imaging evaluations for symptoms like upper abdominal pain or gastrointestinal bleeding (8). Immunohistochemical profiling remains pivotal for diagnosis, with 95% of cases expressing CD117 (KIT protein) and 70% showing CD34 positivity (9). Therefore, in practice, CD117 often considered to be best defining feature of GISTs, while CD34 provides complementary diagnostic value, particularly in CD117-negative cases. Genetics characterization plays a critical role in therapeutic decision-making and prognosis. Approximately 85% of GISTs harbor mutually exclusive activating mutations in *KIT* (exons 9, 11, 13, 17) or *PDGFRA* (exons 12, 18), driving constitutive receptor tyrosine kinase (RTK) activation and tumor progression (3, 10). The remaining 15% lack these canonical mutations and are classified as *KIT/PDGFRA* wild-type (WT) GISTs (11). Notably, recent studies identify *NTRK* gene rearrangement in approximately 16% of *KIT* WT GISTs, expanding the molecular landscape of this subset (12).Serological biomarker detection is also of great significance. Relevant studies have shown that preoperative and postoperative serum CA125 abnormalities can be regarded as independent risk factors for gastrointestinal stromal tumor progression, which is of great value to the comprehensive treatment of patients (13).

For localized and resectable GIST, surgical resection remains the only potentially curative treatment. However, postoperative recurrence occurs in 40 to 90% of patients (14). Neoadjuvant therapy is therefore recommended for patients with large GISTs to reduce tumor volume, minimize intraoperative rupture risk and improve resectability (15). Current National Comprehensive Cancer Network (NCCN) guidelines endorse imatinib for locally advanced or marginally resectable primary GISTs (16). While complete surgical excision combined with adjuvant imatinib constitutes the standard treatment for intermediate- and high-risk GISTs, bulky tumors often necessitate multivisceral resection or functional organ compromise. Preoperative imatinib administration facilitates R0 resection and organ preservation through tumor downsizing (17). Clinical studies demonstrate that neoadjuvant imatinib (400 mg daily) achieves significant tumor regression in massive GISTs (>10 cm), enabling radical resection in most cases (18–21). In the present case, the patient was found to harbor a *KIT* exon 11 deletion (p.K558_V560del) with a 63.80% VAF, achieving PR after imatinib therapy. Beyond tumor downsizing, three phase III clinical trials (ACOSOG Z9001, SSGXVIII/AIO, and EORTC 62024) have confirmed the long-term survival benefits of adjuvant imatinib, the 10-year overall survival rates reached 79% in high-risk cohorts (22–24). Based on this evidence and beyond, current clinical guidelines recommend 3-year adjuvant imatinib (400 mg daily) for high-risk GIST patients carrying imatinib-sensitive *KIT* or *PDGFRA* mutations (25).

Unfortunately, follow-up contrast-enhanced CT on March 28, 2024, revealed disease progression, which indicates an acquired resistance to imatinib. To our knowledge, approximately 50% of GIST patients develop imatinib resistance within 24 months, primarily due to acquired *cis*-mutations in the *KIT* and *PDGFRA*. Common resistance mutations include *KIT* V654A (exon 13), T670I (exon 14), D816V/D820G/N822K/Y823D (exon 17), and *PDGFRA* D842V (exon 18), which likely arise through Darwinian selection under TKI pressure (26, 27). Other drug resistance mechanisms include *KIT* overexpression, activation of downstream and/or alternative pathways, and *BRAF* mutations. Importantly, drug-resistant cells may undergo clonal proliferation, with different metastatic lesions potentially exhibiting distinct mutation profiles. Therefore, ctDNA profiling has emerged as a critical tool for comprehensive resistance mutation analysis, providing essential insights to guide therapeutic decision-making in resistant malignancies (28). In this case, *KIT* mutation and *NTRK3* fusion were identified through plasma ctDNA analysis, and entrectinib 600 mg daily was initiated on March 28, 2024. Subsequent CT results (April 25, 2024) demonstrated partial response (RECIST 1.1), with complete molecular remission (both alterations undetectable, VAF 0%) confirmed at 3 months. While rare in adult malignancies, *NTRK* fusions are hallmark alterations in WT GIST lacking *KIT/PDGFRA/RAS* pathway mutations (29–31). The prototypical *ETV6-NTRK3* fusion, first identified in infantile fibrosarcoma (32), has been reported in <2% of quadruple WT GISTs (31, 33). Brenca et al. proposed that *ETV6-NTRK3* may drive tumorigenesis through IGF1R/IRS1 pathway activation (30). TRK inhibitors (entrectinib/larotrectinib) demonstrate remarkable efficacy across *NTRK*-fusion cancers, with reported response rates exceeding 75% in basket trials (31, 34–37). Notably, all three GIST patients in the larotrectinib NAVIGATE trial achieved >30% tumor shrinkage, including one pathologic complete response (37). The Belgian consensus guidelines now recommend larotrectinib as first-line therapy for *NTRK*-fusion GIST (38). Remarkably, follow-up CT at 1 month (April 25, 2024) showed PR, with complete clearance of both mutations at 3 months. This correlates with emerging evidence that ctDNA dynamics predict clinical outcomes in GIST. Studies demonstrate that ctDNA clearance associates with superior PFS (HR=0.28) and OS (HR=0.19), along with improved ORR (41.7% vs 12.1%) and DCR (97.5% vs 67.2%) (39–41). Our case highlights the clinical utility of serial liquid biopsies for monitoring molecular response and guiding precision therapy.

## Conclusion

While *NTRK* fusions occur at extremely low frequencies (<1%) in GIST, this case highlights the clinical potential of TRK inhibitors (e.g., entrectinib) for overcoming imatinib resistance in molecularly selected patients.Based on the above,the integration of NGS and liquid biopsy has revolutionized the molecular profiling of GIST, enabling the detection of rare mutations and early identification of secondary resistance mechanisms. Although our findings are limited by the short follow-up duration and single-case nature, the observed rapid molecular clearance (VAF 0%) and radiographic response (PR) provide compelling preliminary evidence. These results align with recent basket trials demonstrating >75% response rates to TRK inhibitors in *NTRK*-fusion solid tumors (33–35).

Moving forward, systematic collection of real-world data through multicenter registries is critical to validate the long-term efficacy and safety of this targeted approach. Furthermore, prospective studies should explore optimal sequencing strategies combining TRK inhibitors with other targeted therapies to delay resistance. This paradigm-shifting case underscores the necessity of comprehensive molecular profiling in imatinib-resistant GIST and expands the therapeutic arsenal for precision oncology.

## Data Availability

The datasets presented in this study can be found in online repositories. The names of the repository/repositories and accession number(s) can be found in the article/Supplementary Material.
